# 1-[(3-Methyl­piperidin-1-yl)(3-nitro­phen­yl)meth­yl]naphthalen-2-ol

**DOI:** 10.1107/S1600536810045149

**Published:** 2010-11-10

**Authors:** Jin Mei Chen, Hong Zhao

**Affiliations:** a School of Chemistry and Chemical Engineering, Southeast University, Nanjing 210096, People’s Republic of China

## Abstract

The title compound, C_23_H_24_N_2_O_3_, was synthesized from naphthalen-2-ol, 3-nitro­benzaldehyde and 3-methyl­piperidine. The dihedral angles between the naphthalene system and the nitro­benzene and methyl­piperidine rings are 78.53 (13) and 64.14 (15)°, respectively. The mol­ecular conformation is stabilized by a strong intra­molecular O—H⋯N hydrogen bond.

## Related literature

For applications of naphthalen-2-ol derivatives in catalytic asymmetric synthesis, see: Szatmari & Fulop (2004 ▶). For related structures, see: Zhao & Sun (2005 ▶); Wang & Zhao (2009 ▶); Xiao & Zhao (2010 ▶);
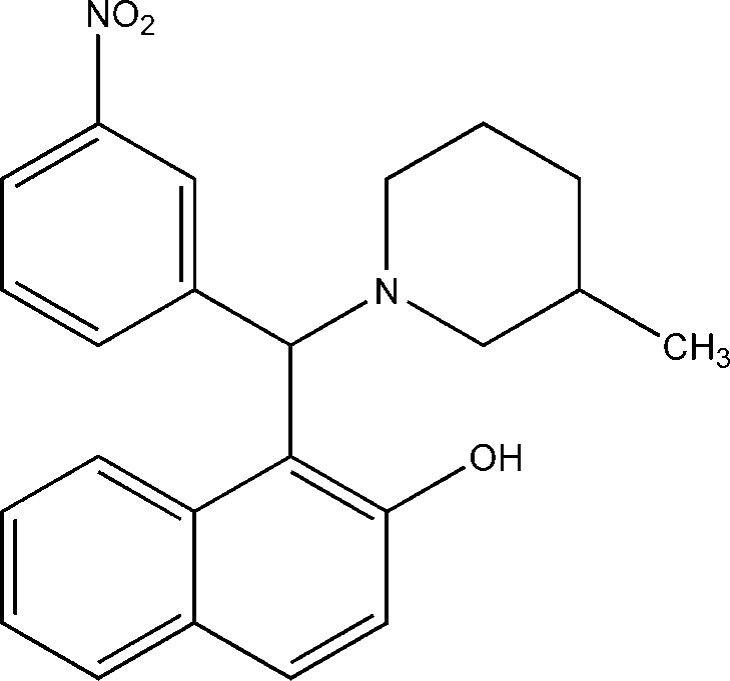


## Experimental

### 

#### Crystal data

C_23_H_24_N_2_O_3_
*M* *_r_* = 376.44
Orthorhombic, 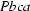

*a* = 11.980 (2) Å
*b* = 10.965 (2) Å
*c* = 30.30 (3) Å
*V* = 3980 (4) Å^3^
*Z* = 8
Mo *K*α radiation
μ = 0.08 mm^−1^
*T* = 295 K
0.25 × 0.22 × 0.18 mm

#### Data collection

Rigaku SCXmini diffractometer
Absorption correction: multi-scan (*CrystalClear*; Rigaku, 2005 ▶) *T*_min_ = 0.982, *T*_max_ = 0.992
34080 measured reflections
3877 independent reflections
2409 reflections with *I* > 2σ(*I*)
*R*_int_ = 0.110

#### Refinement

*R*[*F* ^2^ > 2σ(*F* ^2^)] = 0.083
*wR*(*F* ^2^) = 0.174
*S* = 1.12
3877 reflections
255 parameters
H-atom parameters constrained
Δρ_max_ = 0.14 e Å^−3^
Δρ_min_ = −0.15 e Å^−3^

### 

Data collection: *CrystalClear* (Rigaku, 2005 ▶); cell refinement: *CrystalClear*; data reduction: *CrystalClear*; program(s) used to solve structure: *SHELXS97* (Sheldrick, 2008 ▶); program(s) used to refine structure: *SHELXL97* (Sheldrick, 2008 ▶); molecular graphics: *SHELXTL/PC* (Sheldrick, 2008 ▶); software used to prepare material for publication: *SHELXTL/PC*.

## Supplementary Material

Crystal structure: contains datablocks I, global. DOI: 10.1107/S1600536810045149/bx2327sup1.cif

Structure factors: contains datablocks I. DOI: 10.1107/S1600536810045149/bx2327Isup2.hkl

Additional supplementary materials:  crystallographic information; 3D view; checkCIF report

## Figures and Tables

**Table 1:** Hydrogen-bond geometry (Å, °)

*D*—H⋯*A*	*D*—H	H⋯*A*	*D*⋯*A*	*D*—H⋯*A*
O1—H1⋯N1	0.82	1.86	2.579 (3)	147

**Fig. 1.**
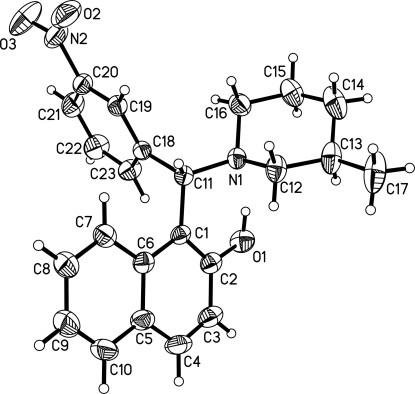
The molecular structure of the title compound, showing the atomic numbering scheme. The displacement ellipsoids are drawn at the 30% probability level.

## supplementary crystallographic information

### Comment

Compounds derived from naphthalen-2-ol have been of great interest in organic chemistry due to their application in catalytic asymmetric synthesis (Szatmari & Fulop, 2004; Zhao & Sun, 2005). As an extension of our work on the structural characterization of naphthol compounds (Wang & Zhao, 2009; Xiao & Zhao, 2010), we report here the structure of (I). In the title compound (Fig. 1) bond lengths and angles have normal values.The dihedral angle between the naphthylen fragment with  the nitrobenzene and methyl piperidine rings are 78.53 (13) and 64.14 (15)° respectively. The molecular conformation is stabilized by one strong intramolecular O—H···N hydrogen bonding (Table 1).

### Experimental

A dry 50 ml flask was charged with 3-nitrobenzaldehyde (10 mmol), naphthalen-2-ol (10 mmol) and 3-methylpiperidine (10 mmol). The mixture was stirred at 100°C for 12 h and then added ethanol (15 ml), after heated under reflux for 1 h, the precipitate was filtrated out and washed with ethanol for three times to give (I). Colourless crystals suitable for X-ray diffraction were obtained by slow evaporation of a dichloromethane solution.

### Refinement

All H atoms were detected in a difference map, but were placed in calculated positions and refined using a riding motion approxmation, with C—H=0.93–0.98 Å, with *U*_iso_(H)=1.2*U*_eq_(C) and *U*_iso_(H_methyl_)=1.2*U*_eq_(C_methyl_); O—H=0.82 Å, with *U*_iso_(H)=1.5*U*_eq_(O).

### Crystal data

**Table:**

C_23_H_24_N_2_O_3_	*F*(000) = 1600
*M**_r_* = 376.44	*D*_x_ = 1.256 Mg m^−^^3^
Orthorhombic, *P**b**c**a*	Mo *K*α radiation, λ = 0.71073 Å
Hall symbol: -P 2ac 2ab	Cell parameters from 4541 reflections
*a* = 11.980 (2) Å	θ = 2.3–27.5°
*b* = 10.965 (2) Å	µ = 0.08 mm^−^^1^
*c* = 30.30 (3) Å	*T* = 295 K
*V* = 3980 (4) Å^3^	Prism, colourless
*Z* = 8	0.25 × 0.22 × 0.18 mm

### Data collection

**Table:**

Rigaku SCXmini diffractometer	3877 independent reflections
Radiation source: fine-focus sealed tube	2409 reflections with *I* > 2σ(*I*)
graphite	*R*_int_ = 0.110
Detector resolution: 13.6612 pixels mm^-1^	θ_max_ = 26.0°, θ_min_ = 2.6°
CCD_Profile_fitting scans	*h* = −14→14
Absorption correction: multi-scan (*CrystalClear*; Rigaku, 2005)	*k* = −13→13
*T*_min_ = 0.982, *T*_max_ = 0.992	*l* = −37→37
34080 measured reflections	

### Refinement

**Table:**

Refinement on *F*^2^	Primary atom site location: structure-invariant direct methods
Least-squares matrix: full	Secondary atom site location: difference Fourier map
*R*[*F*^2^ > 2σ(*F*^2^)] = 0.083	Hydrogen site location: inferred from neighbouring sites
*wR*(*F*^2^) = 0.174	H-atom parameters constrained
*S* = 1.12	*w* = 1/[σ^2^(*F*_o_^2^) + (0.0563*P*)^2^ + 1.4193*P*] where *P* = (*F*_o_^2^ + 2*F*_c_^2^)/3
3877 reflections	(Δ/σ)_max_ < 0.001
255 parameters	Δρ_max_ = 0.14 e Å^−^^3^
0 restraints	Δρ_min_ = −0.15 e Å^−^^3^

### Special details

**Table:**

Geometry. All e.s.d.'s (except the e.s.d. in the dihedral angle between two l.s. planes) are estimated using the full covariance matrix. The cell e.s.d.'s are taken into account individually in the estimation of e.s.d.'s in distances, angles and torsion angles; correlations between e.s.d.'s in cell parameters are only used when they are defined by crystal symmetry. An approximate (isotropic) treatment of cell e.s.d.'s is used for estimating e.s.d.'s involving l.s. planes.
Refinement. Refinement of *F*^2^ against ALL reflections. The weighted *R*-factor *wR* and goodness of fit *S* are based on *F*^2^, conventional *R*-factors *R* are based on *F*, with *F* set to zero for negative *F*^2^. The threshold expression of *F*^2^ > σ(*F*^2^) is used only for calculating *R*-factors(gt) *etc*. and is not relevant to the choice of reflections for refinement. *R*-factors based on *F*^2^ are statistically about twice as large as those based on *F*, and *R*- factors based on ALL data will be even larger.

### Fractional atomic coordinates and isotropic or equivalent isotropic displacement parameters (Å^2^)

**Table:**

	*x*	*y*	*z*	*U*_iso_*/*U*_eq_	
C1	0.7000 (2)	0.9558 (2)	0.65000 (10)	0.0433 (7)	
C2	0.6194 (2)	1.0425 (3)	0.64171 (11)	0.0523 (8)	
C3	0.5315 (3)	1.0634 (3)	0.67144 (13)	0.0659 (9)	
H3	0.4769	1.1206	0.6646	0.079*	
C4	0.5255 (3)	1.0015 (4)	0.70975 (13)	0.0707 (10)	
H4	0.4661	1.0161	0.7288	0.085*	
C5	0.6075 (3)	0.9147 (3)	0.72156 (11)	0.0584 (9)	
C6	0.6961 (2)	0.8920 (3)	0.69134 (10)	0.0478 (7)	
C7	0.7785 (3)	0.8061 (3)	0.70421 (10)	0.0552 (8)	
H7	0.8381	0.7905	0.6854	0.066*	
C8	0.7725 (3)	0.7459 (3)	0.74356 (11)	0.0708 (10)	
H8	0.8279	0.6904	0.7513	0.085*	
C9	0.6840 (3)	0.7671 (4)	0.77209 (12)	0.0820 (12)	
H9	0.6796	0.7246	0.7986	0.098*	
C10	0.6041 (3)	0.8493 (4)	0.76161 (12)	0.0774 (11)	
H10	0.5457	0.8630	0.7812	0.093*	
C11	0.7905 (2)	0.9244 (2)	0.61686 (9)	0.0422 (7)	
H11	0.8033	0.8363	0.6189	0.051*	
C12	0.6680 (3)	0.8576 (3)	0.55808 (11)	0.0599 (9)	
H12A	0.6106	0.8529	0.5806	0.072*	
H12B	0.7044	0.7787	0.5564	0.072*	
C13	0.6143 (3)	0.8861 (3)	0.51400 (13)	0.0728 (11)	
H13	0.5776	0.9657	0.5166	0.087*	
C14	0.7021 (4)	0.8968 (4)	0.47882 (13)	0.0945 (14)	
H14A	0.6680	0.9242	0.4515	0.113*	
H14B	0.7353	0.8175	0.4736	0.113*	
C15	0.7918 (3)	0.9860 (4)	0.49260 (11)	0.0828 (12)	
H15A	0.8513	0.9851	0.4709	0.099*	
H15B	0.7605	1.0676	0.4933	0.099*	
C16	0.8395 (3)	0.9559 (3)	0.53738 (10)	0.0633 (9)	
H16A	0.8776	0.8780	0.5361	0.076*	
H16B	0.8936	1.0176	0.5457	0.076*	
C17	0.5250 (4)	0.7925 (3)	0.50331 (16)	0.1061 (16)	
H17A	0.4876	0.8153	0.4765	0.159*	
H17B	0.4719	0.7892	0.5270	0.159*	
H17C	0.5590	0.7139	0.4996	0.159*	
C18	0.9014 (2)	0.9868 (3)	0.62676 (9)	0.0428 (7)	
C19	1.0000 (2)	0.9224 (3)	0.62142 (9)	0.0472 (7)	
H19	0.9988	0.8410	0.6128	0.057*	
C20	1.0999 (3)	0.9808 (3)	0.62900 (10)	0.0553 (8)	
C21	1.1060 (3)	1.1004 (4)	0.64166 (12)	0.0710 (10)	
H21	1.1746	1.1379	0.6463	0.085*	
C22	1.0079 (3)	1.1635 (3)	0.64731 (13)	0.0748 (10)	
H22	1.0099	1.2448	0.6560	0.090*	
C23	0.9067 (3)	1.1075 (3)	0.64020 (11)	0.0584 (8)	
H23	0.8410	1.1512	0.6445	0.070*	
N1	0.7505 (2)	0.9505 (2)	0.57076 (7)	0.0469 (6)	
N2	1.2039 (3)	0.9098 (4)	0.62304 (11)	0.0788 (9)	
O1	0.61885 (19)	1.11174 (19)	0.60442 (8)	0.0645 (6)	
H1	0.6631	1.0833	0.5865	0.097*	
O2	1.1967 (2)	0.8052 (3)	0.61087 (11)	0.1051 (10)	
O3	1.2920 (2)	0.9589 (3)	0.63113 (13)	0.1305 (14)	

### Atomic displacement parameters (Å^2^)

**Table:**

	*U*^11^	*U*^22^	*U*^33^	*U*^12^	*U*^13^	*U*^23^
C1	0.0363 (15)	0.0400 (15)	0.0536 (18)	−0.0022 (13)	−0.0002 (13)	−0.0087 (13)
C2	0.0470 (18)	0.0457 (17)	0.064 (2)	−0.0034 (15)	−0.0002 (15)	−0.0073 (15)
C3	0.047 (2)	0.066 (2)	0.085 (3)	0.0061 (17)	0.0059 (18)	−0.010 (2)
C4	0.048 (2)	0.084 (3)	0.079 (3)	−0.0049 (19)	0.0190 (18)	−0.019 (2)
C5	0.0454 (19)	0.068 (2)	0.062 (2)	−0.0110 (17)	0.0083 (16)	−0.0132 (17)
C6	0.0440 (17)	0.0445 (16)	0.0550 (19)	−0.0103 (14)	−0.0010 (14)	−0.0083 (14)
C7	0.057 (2)	0.0570 (19)	0.0515 (19)	−0.0080 (16)	0.0011 (15)	−0.0019 (15)
C8	0.072 (2)	0.086 (3)	0.054 (2)	−0.001 (2)	0.0013 (18)	0.0116 (19)
C9	0.080 (3)	0.109 (3)	0.057 (2)	−0.012 (3)	0.004 (2)	0.020 (2)
C10	0.064 (2)	0.110 (3)	0.058 (2)	−0.017 (2)	0.0178 (19)	−0.006 (2)
C11	0.0437 (17)	0.0387 (15)	0.0443 (16)	0.0001 (13)	−0.0026 (13)	−0.0012 (12)
C12	0.067 (2)	0.0439 (17)	0.069 (2)	−0.0014 (16)	−0.0200 (17)	−0.0041 (15)
C13	0.086 (3)	0.0478 (19)	0.085 (3)	0.0037 (19)	−0.040 (2)	−0.0030 (18)
C14	0.134 (4)	0.089 (3)	0.060 (3)	0.004 (3)	−0.028 (3)	−0.002 (2)
C15	0.105 (3)	0.090 (3)	0.053 (2)	−0.001 (2)	−0.009 (2)	0.006 (2)
C16	0.072 (2)	0.067 (2)	0.051 (2)	0.0067 (19)	−0.0002 (17)	−0.0001 (16)
C17	0.115 (4)	0.070 (3)	0.134 (4)	−0.009 (2)	−0.071 (3)	−0.004 (2)
C18	0.0425 (17)	0.0462 (17)	0.0397 (16)	−0.0016 (14)	−0.0008 (13)	−0.0007 (12)
C19	0.0503 (18)	0.0472 (17)	0.0441 (17)	0.0015 (15)	−0.0022 (14)	0.0004 (13)
C20	0.0444 (19)	0.069 (2)	0.0522 (19)	−0.0005 (17)	−0.0015 (15)	0.0060 (16)
C21	0.054 (2)	0.076 (2)	0.083 (3)	−0.022 (2)	−0.0081 (19)	−0.005 (2)
C22	0.071 (3)	0.058 (2)	0.095 (3)	−0.013 (2)	−0.002 (2)	−0.0178 (19)
C23	0.053 (2)	0.0474 (18)	0.075 (2)	−0.0015 (16)	0.0026 (17)	−0.0117 (16)
N1	0.0488 (14)	0.0447 (13)	0.0472 (14)	0.0003 (12)	−0.0068 (12)	−0.0026 (11)
N2	0.0454 (19)	0.101 (3)	0.090 (2)	0.009 (2)	−0.0059 (16)	0.011 (2)
O1	0.0670 (16)	0.0489 (13)	0.0775 (17)	0.0158 (11)	0.0021 (12)	0.0042 (12)
O2	0.0703 (19)	0.106 (2)	0.139 (3)	0.0348 (18)	−0.0131 (17)	−0.027 (2)
O3	0.0440 (17)	0.134 (3)	0.214 (4)	−0.0033 (18)	−0.020 (2)	0.008 (3)

### Geometric parameters (Å, °)

**Table:**

C1—C2	1.379 (4)	C13—H13	0.9800
C1—C6	1.435 (4)	C14—C15	1.511 (5)
C1—C11	1.517 (4)	C14—H14A	0.9700
C2—O1	1.361 (4)	C14—H14B	0.9700
C2—C3	1.404 (4)	C15—C16	1.509 (5)
C3—C4	1.347 (5)	C15—H15A	0.9700
C3—H3	0.9300	C15—H15B	0.9700
C4—C5	1.413 (5)	C16—N1	1.471 (4)
C4—H4	0.9300	C16—H16A	0.9700
C5—C10	1.410 (5)	C16—H16B	0.9700
C5—C6	1.424 (4)	C17—H17A	0.9600
C6—C7	1.419 (4)	C17—H17B	0.9600
C7—C8	1.365 (4)	C17—H17C	0.9600
C7—H7	0.9300	C18—C19	1.386 (4)
C8—C9	1.387 (5)	C18—C23	1.387 (4)
C8—H8	0.9300	C19—C20	1.376 (4)
C9—C10	1.353 (5)	C19—H19	0.9300
C9—H9	0.9300	C20—C21	1.368 (5)
C10—H10	0.9300	C20—N2	1.481 (4)
C11—N1	1.505 (4)	C21—C22	1.375 (5)
C11—C18	1.524 (4)	C21—H21	0.9300
C11—H11	0.9800	C22—C23	1.376 (4)
C12—N1	1.471 (4)	C22—H22	0.9300
C12—C13	1.515 (5)	C23—H23	0.9300
C12—H12A	0.9700	N2—O2	1.207 (4)
C12—H12B	0.9700	N2—O3	1.210 (4)
C13—C14	1.502 (5)	O1—H1	0.8200
C13—C17	1.518 (5)		
			
C2—C1—C6	118.2 (3)	C13—C14—H14A	109.5
C2—C1—C11	122.5 (3)	C15—C14—H14A	109.5
C6—C1—C11	119.3 (2)	C13—C14—H14B	109.5
O1—C2—C1	122.6 (3)	C15—C14—H14B	109.5
O1—C2—C3	116.0 (3)	H14A—C14—H14B	108.1
C1—C2—C3	121.4 (3)	C16—C15—C14	112.1 (3)
C4—C3—C2	120.7 (3)	C16—C15—H15A	109.2
C4—C3—H3	119.6	C14—C15—H15A	109.2
C2—C3—H3	119.6	C16—C15—H15B	109.2
C3—C4—C5	121.3 (3)	C14—C15—H15B	109.2
C3—C4—H4	119.3	H15A—C15—H15B	107.9
C5—C4—H4	119.3	N1—C16—C15	110.6 (3)
C10—C5—C4	122.7 (3)	N1—C16—H16A	109.5
C10—C5—C6	119.0 (3)	C15—C16—H16A	109.5
C4—C5—C6	118.3 (3)	N1—C16—H16B	109.5
C7—C6—C5	117.3 (3)	C15—C16—H16B	109.5
C7—C6—C1	122.8 (3)	H16A—C16—H16B	108.1
C5—C6—C1	120.0 (3)	C13—C17—H17A	109.5
C8—C7—C6	121.7 (3)	C13—C17—H17B	109.5
C8—C7—H7	119.2	H17A—C17—H17B	109.5
C6—C7—H7	119.2	C13—C17—H17C	109.5
C7—C8—C9	120.2 (4)	H17A—C17—H17C	109.5
C7—C8—H8	119.9	H17B—C17—H17C	109.5
C9—C8—H8	119.9	C19—C18—C23	118.8 (3)
C10—C9—C8	120.4 (3)	C19—C18—C11	119.5 (3)
C10—C9—H9	119.8	C23—C18—C11	121.7 (3)
C8—C9—H9	119.8	C20—C19—C18	119.0 (3)
C9—C10—C5	121.4 (3)	C20—C19—H19	120.5
C9—C10—H10	119.3	C18—C19—H19	120.5
C5—C10—H10	119.3	C21—C20—C19	122.6 (3)
N1—C11—C1	110.1 (2)	C21—C20—N2	119.6 (3)
N1—C11—C18	112.0 (2)	C19—C20—N2	117.8 (3)
C1—C11—C18	113.0 (2)	C20—C21—C22	118.1 (3)
N1—C11—H11	107.1	C20—C21—H21	120.9
C1—C11—H11	107.1	C22—C21—H21	120.9
C18—C11—H11	107.1	C21—C22—C23	120.6 (3)
N1—C12—C13	111.9 (3)	C21—C22—H22	119.7
N1—C12—H12A	109.2	C23—C22—H22	119.7
C13—C12—H12A	109.2	C22—C23—C18	120.8 (3)
N1—C12—H12B	109.2	C22—C23—H23	119.6
C13—C12—H12B	109.2	C18—C23—H23	119.6
H12A—C12—H12B	107.9	C12—N1—C16	109.6 (2)
C14—C13—C12	110.1 (3)	C12—N1—C11	109.0 (2)
C14—C13—C17	113.3 (4)	C16—N1—C11	114.5 (2)
C12—C13—C17	110.3 (3)	O2—N2—O3	123.2 (4)
C14—C13—H13	107.6	O2—N2—C20	118.5 (3)
C12—C13—H13	107.6	O3—N2—C20	118.3 (4)
C17—C13—H13	107.6	C2—O1—H1	109.5
C13—C14—C15	110.6 (3)		
			
C6—C1—C2—O1	176.9 (3)	C12—C13—C14—C15	−52.7 (4)
C11—C1—C2—O1	−4.2 (4)	C17—C13—C14—C15	−176.8 (3)
C6—C1—C2—C3	−3.8 (4)	C13—C14—C15—C16	53.0 (4)
C11—C1—C2—C3	175.1 (3)	C14—C15—C16—N1	−56.1 (4)
O1—C2—C3—C4	−178.7 (3)	N1—C11—C18—C19	−95.4 (3)
C1—C2—C3—C4	2.0 (5)	C1—C11—C18—C19	139.5 (3)
C2—C3—C4—C5	0.7 (5)	N1—C11—C18—C23	83.3 (3)
C3—C4—C5—C10	179.3 (3)	C1—C11—C18—C23	−41.7 (4)
C3—C4—C5—C6	−1.4 (5)	C23—C18—C19—C20	−0.9 (4)
C10—C5—C6—C7	−1.8 (4)	C11—C18—C19—C20	177.8 (3)
C4—C5—C6—C7	178.9 (3)	C18—C19—C20—C21	0.0 (5)
C10—C5—C6—C1	178.8 (3)	C18—C19—C20—N2	180.0 (3)
C4—C5—C6—C1	−0.5 (4)	C19—C20—C21—C22	0.6 (5)
C2—C1—C6—C7	−176.4 (3)	N2—C20—C21—C22	−179.4 (3)
C11—C1—C6—C7	4.7 (4)	C20—C21—C22—C23	−0.2 (6)
C2—C1—C6—C5	3.0 (4)	C21—C22—C23—C18	−0.7 (6)
C11—C1—C6—C5	−175.9 (2)	C19—C18—C23—C22	1.3 (5)
C5—C6—C7—C8	1.1 (4)	C11—C18—C23—C22	−177.5 (3)
C1—C6—C7—C8	−179.5 (3)	C13—C12—N1—C16	−60.4 (3)
C6—C7—C8—C9	0.4 (5)	C13—C12—N1—C11	173.6 (3)
C7—C8—C9—C10	−1.3 (6)	C15—C16—N1—C12	58.8 (3)
C8—C9—C10—C5	0.6 (6)	C15—C16—N1—C11	−178.4 (3)
C4—C5—C10—C9	−179.8 (4)	C1—C11—N1—C12	−72.9 (3)
C6—C5—C10—C9	1.0 (5)	C18—C11—N1—C12	160.5 (2)
C2—C1—C11—N1	−26.7 (3)	C1—C11—N1—C16	164.0 (2)
C6—C1—C11—N1	152.2 (2)	C18—C11—N1—C16	37.3 (3)
C2—C1—C11—C18	99.5 (3)	C21—C20—N2—O2	−178.3 (4)
C6—C1—C11—C18	−81.7 (3)	C19—C20—N2—O2	1.7 (5)
N1—C12—C13—C14	57.6 (4)	C21—C20—N2—O3	2.6 (5)
N1—C12—C13—C17	−176.7 (3)	C19—C20—N2—O3	−177.4 (3)

### Hydrogen-bond geometry (Å, °)

**Table:**

*D*—H···*A*	*D*—H	H···*A*	*D*···*A*	*D*—H···*A*
O1—H1···N1	0.82	1.86	2.579 (3)	147
